# Structural Learning in Autistic and Non-Autistic Children: A Replication and Extension

**DOI:** 10.1007/s10803-024-06486-0

**Published:** 2024-09-13

**Authors:** Svenja Oestreicher, Dermot M. Bowler, Claire T. Derwent, Sebastian B. Gaigg, Veit Roessner, Nora Vetter, Theresia Volk, Nicole Beyer, Melanie Ring

**Affiliations:** 1 Department of Child and Adolescent Psychiatry, Medical Faculty, University Hospital Carl Gustav Carus, Technische Universität Dresden, Fetscherstraße 74, 01307 Dresden, Germany; 2 Autism Research Group, Department of Psychology, City, University of London, London, UK; 3 Medical School Berlin, Department of Psychology, Berlin, Germany

**Keywords:** Autism, Children, Hippocampus, Structural and configural learning, Executive functions

## Abstract

The hippocampus is involved in many cognitive domains which are difficult for autistic individuals. Our previous study using a Structural Learning task that has been shown to depend on hippocampal functioning found that structural learning is diminished in autistic adults (Ring et al., 2017). The aim of the present study was to examine whether those results can be replicated in and extended to a sample of autistic and non-autistic children. We tested 43 autistic children and 38 non-autistic children with a subsample of 25 autistic and 28 non-autistic children who were well-matched on IQ. The children took part in a Simple Discrimination task which a simpler form of compound learning, and a Structural Learning task. We expected both groups to perform similarly in Simple Discrimination but reduced performance by the autism group on the Structural Learning task, which is what we found in both the well-matched and the non-matched sample. However, contrary to our prediction and the findings from autistic adults in our previous study, autistic children demonstrated a capacity for Structural Learning and showed an overall better performance in the tasks than was seen in earlier studies. We discuss developmental differences in autism as well as the role of executive functions that may have contributed to better than predicted task performance in this study.

Worldwide, about 1% of children meet criteria for an Autism Spectrum Disorder (ASD) (Zeidan et al., 2022). In addition to the core features, ASD is associated with difficulties in behavioral and cognitive domains, such as spatial memory and spatial navigation (Li et al., 2021; Lind et al., 2014; Vogan et al., 2019; Zhang et al., 2020), learning (Marsh et al., 2013; Mayes & Calhoun, 2007; O’Brien & Pearson, 2004), episodic memory and episodic future thinking (Desaunay et al., 2020; Lind et al., 2013, 2014; Marini et al., 2019; Naito et al., 2020). In an attempt to characterize the autistic clinical picture at a neural level, researchers such as Boucher and Warrington (1976), Bowler et al. (2011), DeLong (1992) and Waterhouse et al. (1996) have advocated hippocampal functioning as a possible underlying mechanism. Other researchers outside the autism field have implicated hippocampal and medial temporal lobe processes in learning, memory, navigation (O’Keefe & Nadel, 1978; Rudy & Sutherland, 1995; Sutherland & Rudy, 1989) as well as in social interaction and communication (Rubin et al., 2014; Schiller et al., 2015). However, the role of atypical hippocampal functioning in the etiology of ASD needs further empirical confirmation using tasks that have been shown to depend crucially on intact hippocampal functioning.

In animal lesion studies, damage to the medial temporal lobe and the hippocampus as well as the amygdalohippocampal complex resulted in e.g., memory impairment, socio-emotional impairment such as social withdrawal and lack of interest in social interactions, and repetitive behaviors in the animals similar to the behavioral difficulties seen in autism in humans (Bachevalier, 1994, 1996). Later work (Vargha-Khadem, Gadian, Watkins et al., 1997) has shown that the difficulties resulting from early hippocampal damage relate to episodic memory, which involves the flexible combination and re-combination of elements of experience and not to social emotional difficulties. Yet Bowler et al. (2011) have argued that the social and memory difficulties experienced by autistic individuals may also stem at least in part from a common set of cognitive processes involving flexible thinking that are thought to implicate fronto-hippocampal mechanisms.

Findings from human studies comparing hippocampal volume and metabolism in autistic and non-autistic individuals are inconsistent. Some studies report no differences in volume and metabolism between groups (Haznedar et al., 2000), others report subtle reduction of hippocampal volume in autistic people (Nicolson et al., 2006) and still others report an increased volume of the hippocampus in autistic individuals (Schumann et al., 2004). These findings suggest that the clinical picture of ASD may not solely be associated with structural abnormalities in a specific region, such as the hippocampus, but rather with functional atypicalities in these regions or connectivity with other brain regions such as the frontal lobe executive system (Ellis Weismer et al., 2018; Friedman & Sterling, 2019; Hill, 2004; Hosenbocus & Chahal, 2012). Studies of connectivity between the hippocampus and other brain regions examined in functional imaging studies using tasks related to hippocampal functioning have also showed inconsistent results ranging from no evidence for ASD-related hippocampal atypicalities during relational reasoning in a transitive inference task (Solomon et al., 2015) to a reduced hippocampal connectivity in autistic people during memory retrieval of previously learned object features (Cooper et al., 2017).

These inconclusive findings may indicate that the experimental tasks used in these investigations are not specifically sensitive to hippocampal functioning and might instead depend also on other brain regions, such as the prefrontal cortex (PFC, (Zhang et al., 2022). Inconsistencies might also result from autistic participants’ relying on compensatory mechanisms which mask underlying difficulty (Livingston & Happé, 2017). Therefore, it is necessary to look to conceptualizations of hippocampal processes that are specified in a more abstract and general level, that are known to operate across functional domains and that might be less likely to be compensated for. One such conceptualization that has been shown to be specifically sensitive to hippocampal functioning is *Configural Learning*, which involves the binding of stimulus elements to form unique arrays (an everyday example would be binding knowledge of a particular person and a specific place into a place-person association connected to a unique episode). *Structural Learning* (Aggleton et al., 2007, 2009, 2010; Sanderson et al., 2006) is a more specific form of configural learning that additionally requires the binding of the relationships among stimulus elements, be they spatial (e.g. element A has to be to the left, rather to the right of element B or temporal (element A must occur *before* (rather than after) element B. This process enables us to distinguish between similar stimuli and situations and thus enables us to orient ourselves successfully in the surrounding spatial and social world. For example, we learn that certain behaviors can facilitate social belonging to one group of people in a certain social context and lead to rejection in another social context or by another group of people. The clinical features of ASD such as difficulties with episodic memory and with social interactions suggest autistic individuals should also show atypical Structural Learning compared to non-autistic individuals.

Sanderson et al. (2006) developed an experimental paradigm involving four different visual discrimination tasks including one involving Structural Learning to examine the consequences of hippocampal lesions on rats’ performance on the tasks (see Fig. 1 for examples of the stimuli). In all four tasks, the rats had to learn to discriminate between pairs of stimuli before and after a hippocampal or sham lesion by having to find a submerged platform in a water tank that was below one of two presented stimuli). In *Simple Discrimination*, a simpler form of learning not requiring Configural Learning, rats had to discriminate between two simple images. Three other tasks (Structural Learning, Transverse Patterning and Biconditional Discrimination) tested the more complex Configural Learning. Only the *Structural Learning* task is of relevance for the current project. In this task, rats had to discriminate between pairs of compound images that were made up of simple stimuli that were white, black, or striped and that were mirror images of each other (see Column II, Lines A, B & C of Fig. 1). The discrimination between these mirror images required rats to bind the component stimuli of an image and represent their specific spatial configuration. Sanderson et al. (2006) found that hippocampal lesions impaired the ability of Structural Learning but not the ability to learn and perform the Simple Discrimination pointing to the likely role of the hippocampus in structural learning.

**Fig. 1:**
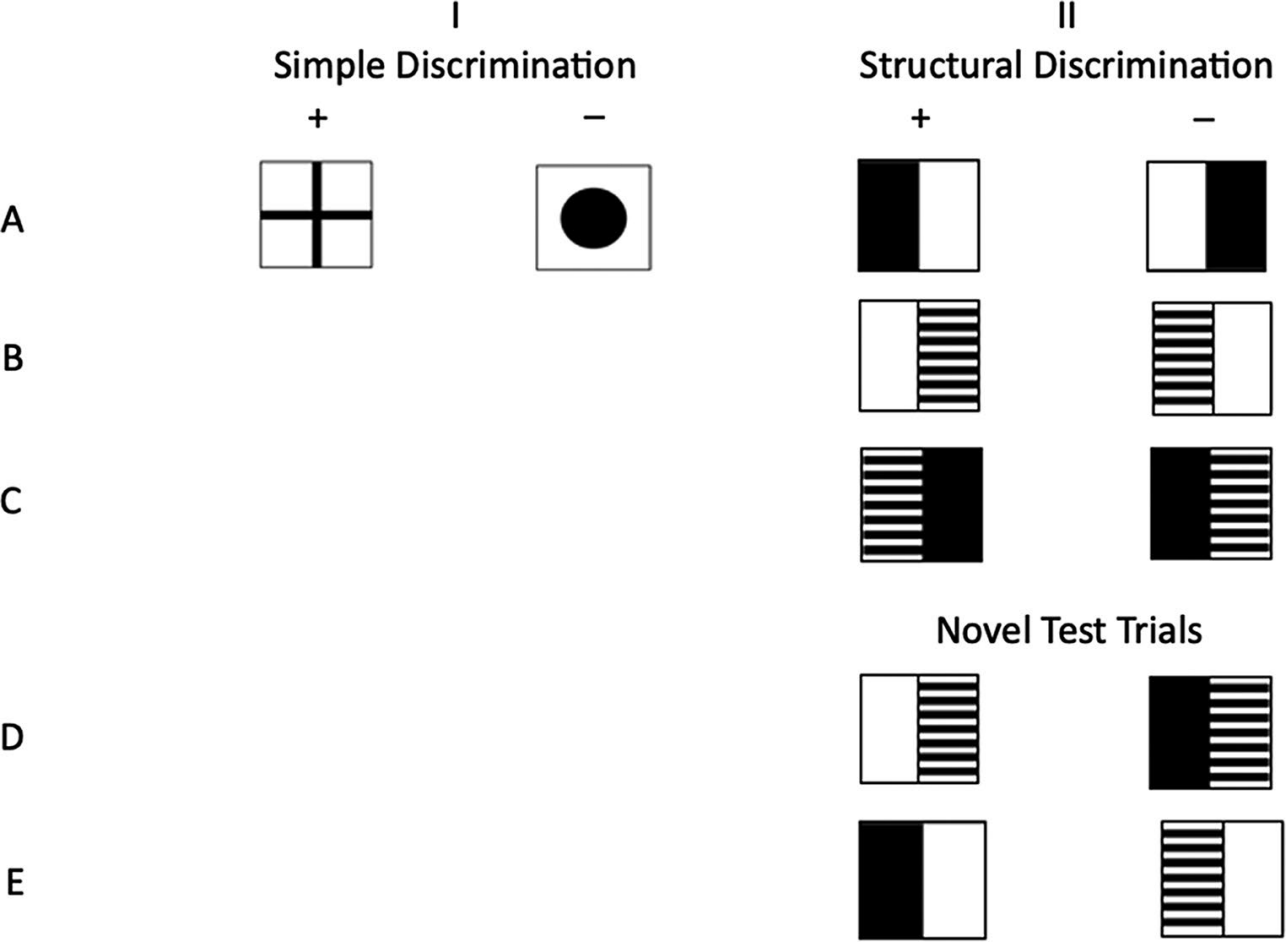
Examples of stimuli for Simple Discrimination (Column I), which were presented in Blocks 1 to 5. Examples of stimuli for Structural Learning (Column II) with mirror image test trials, which were presented in Blocks 1 to 5 (Lines **A** to **C**), and re-paired stimuli test trials, which were presented in Block 5 only (Lines **D** and **E**). Stimuli presented below the plus sign are reinforced in the example (Figure provided by courtesy of Ring et al., 2017)

Ring et al. (2017) adapted the Simple Discrimination and Configural Learning tasks from Sanderson et al. (2006) for use in humans to examine whether autistic adults experience specific difficulties with Structural Learning, but not with tasks of other forms of Configural Learning, like Biconditional Discrimination, or Transverse Patterning, or in Simple Discrimination (see Fig. 1). They tested groups of 19 autistic and 19 non-autistic adults aged in their early 40’s using pairs of visual stimuli similar to those illustrated in Fig. 1 presented on a touch-sensitive screen. Participants were given minimal instructions (‘pick the correct image’) and were provided with smiling or frowning faces as feedback. Ring et al. found a significantly lower performance in autistic compared to non-autistic adults on the Structural Learning task, which was not attributable to problems with executive functions as measured by the Color Trials Test (CTT; D’Elia et al., 1996), or the ability to learn Simple Discriminations. Importantly, autistic adults did not acquire Structural Learning but rather used some other learning strategy to solve the task as indicated by chance performance in their worst discrimination in the task. Furthermore, the authors added an additional test of Structural Learning to the final block of the task by presenting the *studied* mirror image pairs, presented earlier in the task intermixed with *re-paired* image pairs, consisting of the previously seen images, which were rearranged into new non-mirror image pairs (see Fig. 1, Column II lines D and E). Ring et al. assessed ratio scores setting performance on re-paired trials in relation to performance on studied trials (re-paired/(re-paired + studied). The researchers assumed that reduced learning of the structural arrangement of the mirror images presented in the Structural Learning task would lead to a better performance on re-paired trials and higher ratio scores for autistic compared to non-autistic adults, which is what they found. Taken together, the findings of Ring et al. (2017) and of Sanderson et al. (2006) are consistent with atypical hippocampal functioning.

Derwent (2018) examined whether autistic children had reduced Structural Learning by testing autistic and non-autistic children aged seven to sixteen years on the Simple Discrimination Structural Learning tasks used by Ring et al. (2017). No differences in performance between autistic and non-autistic children were found in either task, regardless of whether the stimuli were studied or re-paired. However, unlike non-autistic children, autistic children performed at chance in the re-paired trials of the Structural Learning task. These results are inconsistent with those of Ring et al. (2017). However, methodological issues in the study of Derwent (2018), such as the fact that the two groups of children were not matched on gender, chronological age, or Full-scale Intelligence Quotient (FIQ), limit any comparison with the earlier Ring et al. (2017) study. The present study aimed to examine whether the findings of Ring et al. (2017) could be replicated in a well-matched sample of autistic and non-autistic children, when tested on the Simple and Structural Learning tasks from Ring et al. (2017). We expected that:

1. autistic compared to non-autistic children would show lower Structural Learning,
2. autistic and non-autistic children would not differ in their learning performance on the Simple Discrimination task,
3. the reduced Structural Learning of autistic children would not be attributable to difficulties in executive functions, measured by the executive total score of the parent version of the Behavior Rating Inventory of Executive Function (BRIEF; Gioia et al., 2000),
4. autistic compared to non-autistic children would not demonstrate Structural Learning capacity and.
5. autistic compared to non-autistic children would show higher performance on re-paired as opposed to studied trials.

## Materials and Methods

### Participants

The sample size was based on Ring et al. (2017), who tested 19 autistic and 19 non-autistic adults in the Structural Learning task. Therefore, the goal was to include at least 20 autistic and 20 matched non-autistic children. Overall, 43 autistic and 38 non-autistic children were tested. Of these, seven autistic and one non-autistic participant were excluded as outliers as they performed at chance in Block 5 of Simple Discrimination task assuming that these individuals showed a more general difficulty with learning rather than structural learning. The exclusion resulted in a total sample of 36 autistic and 37 non-autistic children (detailed information about this sample can be found in the Appendix/ Supplementary Materials). Due to a trend difference between the groups in IQ (*U* = 510.00, *p* = .086, *r* = .20), a sub-sample of well-matched autistic and non-autistic groups was created. Groups in this sub-sample were matched on gender and chronological age and participants in each group were matched individually on Full-scale Intelligence Quotient (FIQ) or General Ability Index (GAI) in the case of an inhomogeneous intelligence profile (in both cases +/- 10 IQ points difference at maximum) as measured by the fifth version of the Wechsler Intelligence Scale for Children (WISC-V; Petermann, 2017). This resulted in 28 matched pairs of children and a total sample size of 56 children. Similarly, as above, a further three autistic participants were excluded as outliers as they performed at chance on the Simple Discrimination task Block 5 resulting in a final sample size of 25 autistic and 28 non-autistic children. Groups did not differ significantly in gender (χ^2^ = 1.86, *p* = .492, Φ = 0.19), chronological age and FIQ/GAI (see Table 1).

**Table 1:** Participant characteristics for the autistic and non-autistic groups

	Autistic (25 m, 0f)	Non-autistic (26 m, 2f)			
	*M (SD)*	*M (SD)*	Test value	*p*-value	Effect size
Age (years)	11.16 (1.84) (range 8–14 y)	11.07 (1.84) (range 8–14 y)	*U* = 347.00	.960	*r* = .01
FIQ / GAI	107.00 (16.24)	106.36 (14.11)	*t*(51) = 0.15	.878	Cohen’s *d* = 0.04

*Note* FIQ: Full-scale Intelligence Quotient, GAI: General Ability Index (in case of an inhomogeneous intelligence profile)

Participants were all native German speakers. They were recruited as part of a larger study at the Clinic for Child and Adolescent Psychiatry and -Psychotherapy (KJP) at the University Hospital Dresden aiming to improve the well-being of autistic children through a diet rich in coenzyme Q10. Autistic children were patients at the Autism center of KJP, University Hospital Dresden and were recruited through telephone calls, e-mails, their therapists, and flyers at the hospital. Non-autistic children were recruited through the test person database of the KJP, University Hospital Dresden. In addition, the study was advertised on the website of the KJP, University Hospital Dresden and schools were contacted and asked to hand out flyers to the children.

Inclusion criteria for autistic children were a chronological age of six to fourteen years and a clinical diagnosis of F84.0 (Autistic disorder), F84.1 (Atypical autism) or F84.5 (Asperger’s syndrome) according to the International Statistical Classification of Diseases and Related Health Problems – Tenth Edition (ICD-10, (World Health Organization, 2004). Autistic children received their clinical diagnosis prior participation based on the gold standard for autism diagnosis, i.e., the German versions of Autism Diagnostic Observation Schedule – Version 2 (ADOS-2; German translation: Poustka et al., 2015) and the Autism Diagnostic Interview– Revised (ADI-R; German translation: Bölte et al., 2006). Since autism is often associated with comorbid psychiatric disorders and the intake of psychotropic medication, these were not an exclusion criterion in the autistic group. Clinical characteristics of the autistic group are presented in Table 2. Due to Q10 intake, a soy allergy or treatment with thyroxine were exclusion criteria in this group.

**Table 2:** Clinical characteristics of the autistic group (*N* = 25)

Continuous variables	M	± SD
*ADOS-2*		
Social Affect	8.24	4.59
Restrictive and Repetitive Behaviors	1.68	2.64
Total Score	9.60	5.16
Comparative Value	5.44	2.67
*ADI-R*		
Social Interaction	14.88	5.31
Communication	11.42	3.65
Restrictive and Repetitive Behaviors	3.17	1.93
Categorial variables	*n*	%
*ICD-10 ASD Diagnoses*		
F84.0 (Autistic disorder)	8	32.00
F84.1 (Atypical autism)	5	20.00
F84.5 (Asperger’s syndrome)	12	48.00
*ICD-10 Co-occurring Diagnoses*		
F80.3 (Acquired aphasia with epilepsy)	1	4.00
F81.2 (Mathematics disorder)	1	4.00
F82 (Specific developmental disorder of motor function)	2	8.00
F83 (Mixed specific developmental disorders)	2	8.00
F90.0 (Attention deficit hyperactivity disorder)	2	8.00
*Intake of Psychotropic Medication*		
Lisdexamfetamine	1	4.00

*Note* ADOS-2: Autism Diagnostic Observation Schedule – Version 2, ADI-R: Autism Diagnostic Interview– Revised, ICD-10: International Statistical Classification of Diseases and Related Health Problems – Tenth Edition

For the non-autistic group, inclusion criteria were a chronological age of six to fourteen years and the absence of psychiatric disorders which was measured with a structured diagnostic interview. Based on this, four recruited non-autistic children were excluded from the study. None of the included non-autistic children reported taking medication regularly. Children in both groups were excluded if they had somatic or neurological diseases affecting the nervous system or were in an acute inflammatory state.

All participants were reimbursed for their time with 10 Euro per hour and their travel costs were paid for. This study was discussed and approved by the ethics committee of the University Hospital Dresden (Processing number: EK 427,102,017) and applies to the guidelines of Helsinki. There was no community involvement in the reported study.

### Materials

The materials and procedures of the Simple Discrimination and Structural Learning tasks were adopted from Ring et al. (2017). They involved minimal verbal instructions. Black and white images were presented on a touch-sensitive tablet-screen. A detailed overview of the stimuli and reinforcement contingencies is provided by Ring et al. (2017) in their supplemental materials. For examples of the stimuli, see Fig. 1.

The parent version of the Behavior Rating Inventory of Executive Function (BRIEF; Gioia et al., 2000) was used to determine the extent of executive difficulties in everyday behavior of the children.

### Procedure

In the Simple Discrimination and Structural Learning tasks from Ring et al. (2017) black-white images were presented on the screen (see Fig. 1). Touching one of the images resulted in the presentation of a smiling or frowning cartoon face. The facial expression of the face provided feedback on whether the selected image was correct or incorrect. Participants were instructed to select the correct image and to learn from the feedback given.

In the Simple Discrimination task, one pair of stimuli images of distinct patterns (cross, circle) was presented in all five blocks (see Fig. 1, Column I). The distinction between these stimuli required a simple discrimination.

In the Structural Learning task, participants were presented with three pairs of mirror images, compounded out of simple white, black or striped stimuli, in all five blocks (see Fig. 1, Column II, Lines A to C). Participants needed to learn the spatial configuration of the parts of the images. For example, they had to learn that an image with a black and white area was a correct image only if the black area was on the left of the white area. Successful distinction between these stimuli implied a capacity for Structural Learning.

The tasks consisted of five blocks. In the Simple Discrimination task, a pair of images was introduced in Block 1. This pair was presented in each of the five blocks. In the Structural Learning task, one pair of images was introduced in Block 1, a second one in Block 2 and a third one in Block 3. Once an image pair was introduced, it was presented in each subsequent block.

In Blocks 4 and 5, all pairs were presented intermixed. In each block, the stimulus pairs were presented until participants either demonstrated reliable acquisition of the stimulus contingencies with an accuracy of 50% in the Simple Discrimination task and 80% in the Structural Learning task, or this learning criterion has not been met within three repetitions of the trials of the block. As soon as the learning criterion was reached or not reached after three repetitions of the trials of the block, the next block was presented. This means that participants who did not reach criterion after three repetitions of a block were not excluded from the experiment. After each block, there was a brief pause. Participants continued by touching a pause button on the screen.

In Block 5 of the Structural Learning task, previously studied stimuli were intermixed with re-paired stimuli (see Fig. 1, Column II, Lines D and E). The re-paired stimuli consisted of the previously presented mirror images assembled into new pairs. For example, the previously presented pairs of black/white vs. white/black and white/striped vs. striped/white were recombined in this block to black/white vs. striped/white or white/striped vs. white/black. The distinction between these re-paired stimuli also required Structural Learning. Learning performance in these trials showed whether participants had fully learned the structural arrangement of the stimuli, because what has been learned had to be transferred to new stimulus combinations and could not be solved through simpler learning processes.

### Statistical Analysis

Participants were excluded for all analyses if they showed an average accuracy of < 1 in the Simple Discrimination trials of Block 5. In addition to the analysis of the data of the matched participants which can be found in the Results section of the paper below, the findings for all the tested participants can be found in the Appendix/ Supplementary Materials.

The data for the difference hypotheses were analyzed in IBM SPSS Statistics version 28 (Statistical Package for Social Science IBM^®^). The data for the equivalence hypothesis were analyzed in DATAtab (DATAtab Team, 2023). For analyses in SPSS, the respective standard methods were used and if the requirements for a method were not met, a corresponding, robust alternative method was used instead, if there was one. Nominal data were analyzed with a Chi-square test. In case requirements were not met, the results of the exact Fisher test are reported. Interval data were analyzed with bivariate correlations, t-tests, repeated measures ANOVAs, and regression analyses. If requirements for the t-test were not met, the data were analyzed with the Mann-Whitney U-test or the Wilcoxon test. In case the sphericity assumption was violated in ANOVAs, the Greenhouse Geissler correction was used. For equivalence testing in DATAtab, the TOST procedure was used, consisting of two one-sided t-tests. Assuming that deviations up to 10% in the performance of the groups were practically irrelevant, the equivalence bounds were set on ± 0.1 (Δ_L_ = -0.1; Δ_U_ = 0.1). The significance level of the error probability was set at α = 5% for all tests. A *p*-value below 0.05 was considered as significant. As measures of effect size, Cohen’s *d* with its 95% confidence intervals and Partial eta squared for the standard methods, and Phi and Pearson correlation coefficients for the alternative methods are reported.

## Results

### Simple Discrimination Task

**Fig. 2:**
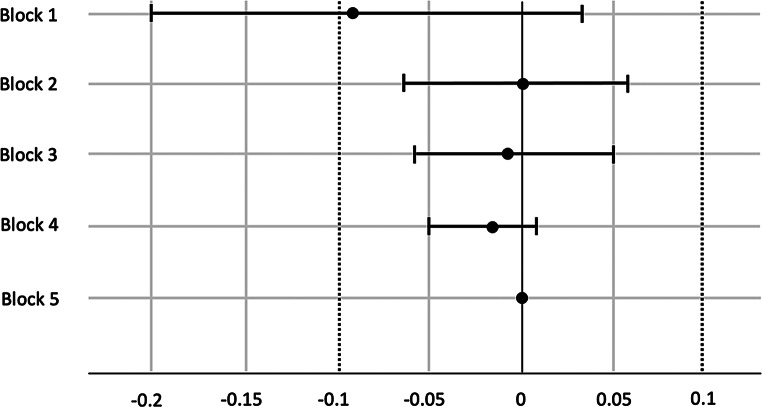
Simple Discrimination mean differences between the autistic and non-autistic groups in Blocks 1 to 5 of the task with 90% confidence intervals

The data are presented in Fig. 2. In Block 1, the TOST procedure yielded a nonsignificant result for the test against Δ_L_, *t*(51) = 0.21, *p* = .417, and a significant result for the test against Δ_U_,*t*(51) = 2.75, *p* = .004. Therefore, the equivalence test was nonsignificant (*p* = .417), indicating that the groups cannot be considered equivalent in Block 1. In Blocks 2 to 5, the TOST procedure yielded significant results for the tests against Δ_L_, all *t*(51) ≥ 2.58, all *p* ≤ .006, and significant results for the tests against Δ_U_, all *t*(51) ≥ 2.82, all *p* ≤ .003. Therefore, the equivalence tests were significant, all *p* ≤ .006, indicating equivalent groups in Blocks 2 to 5 of the task.

### Structural Learning Task

#### Learning

**Fig. 3:**
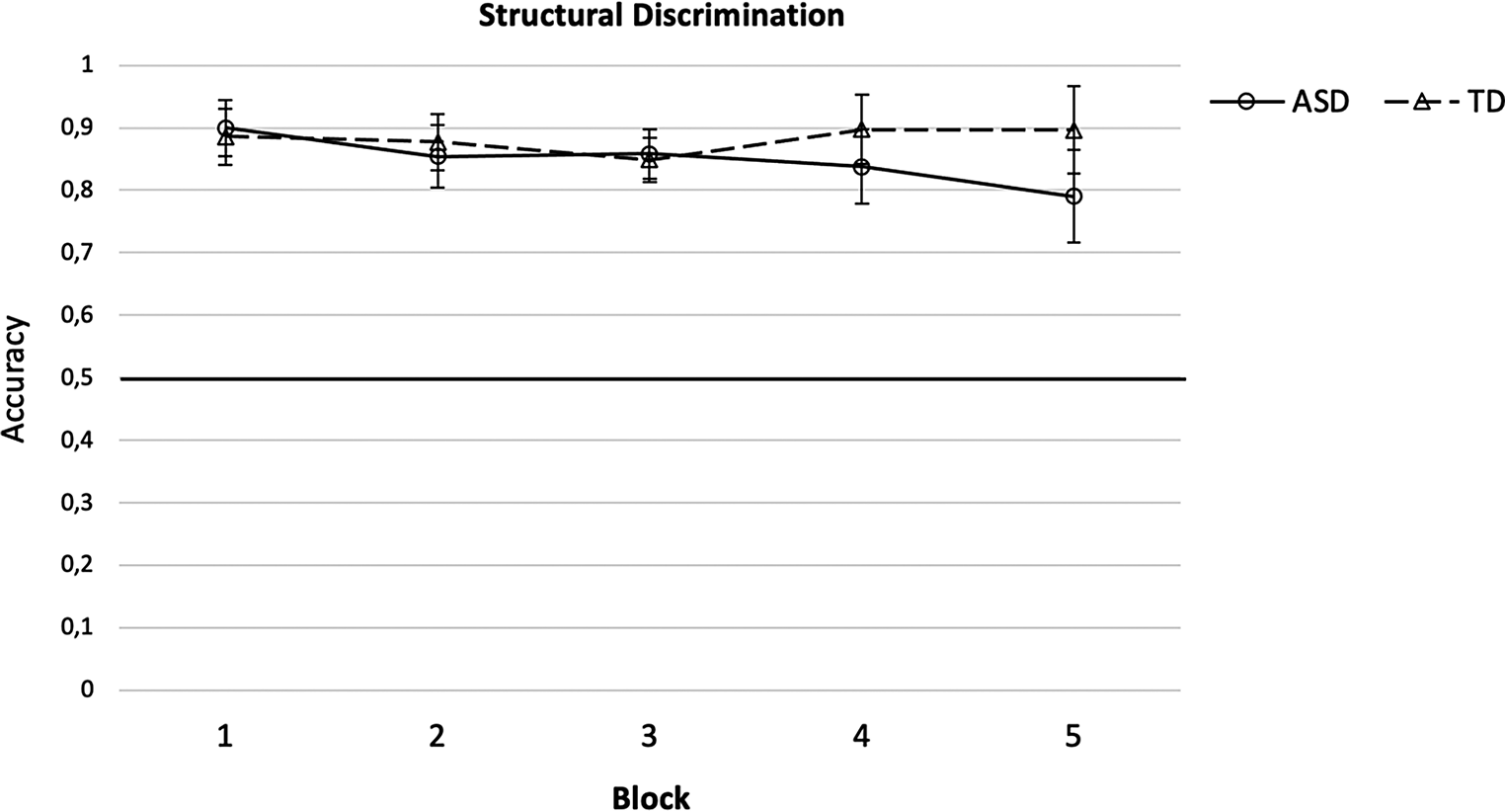
Structural Learning accuracy scores with 95% confidence intervals for the autistic (ASD) and non-autistic (typically developing - TD) groups in Blocks 1 to 5 of the task

The data are presented in Fig. 3. Performance for both groups (autistic – ASD, non-autistic – typically developing *-* TD) was significantly above chance in all five blocks, all *z* ≥ 4.12, all *p* < .001, all *r* ≥ .82. A 2 (Group [ASD, TD]) x 5 (Block [1, 2, 3, 4, 5]) repeated measures ANOVA revealed a significant interaction of Group*Block, *F*(2.77) = 3.14, *p* = .031, η_p_^2^ = 0.06, GGC. Groups differed significantly in their performance in Block 5, *U* = 212.50, *p* = .006, *r* = .34, but not in Blocks 1 to 4, all *U* ≥ 267.00, all *p* ≥ .063, all *r* ≤ .21, indicating a similar performance in both groups in Blocks 1 to 4 followed by an opposite performance in Block 5 with the TD group showing an improvement and the autistic group showing a drop in performance. The main effects of Group, *F*(1) = 1.60, *p* = .212, η_p_^2^ = 0.03, and Block, *F*(2.77) = 1.72, *p* = .170, η_p_^2^ = 0.03, GGC, were not significant.

Block 4 presents the most rigorous test of Structural Learning with all three image pairs studied in Blocks 1 to 4 presented to the participants. To test whether participants of both groups learned all three or only one or two out of three image pairs, separately for each participant, the respective *worst*, *middle* and *best* image pair were identified and performance on these image pairs was compared to chance level. The data are presented in Table 3. Participants in both groups performed significantly better than chance on all three discriminations, all *z* ≥ 3.32 *p* < .001, all *r* ≥ .66, indicating that participants in both groups demonstrated Structural Learning capability.

**Table 3:** Means and standard deviations of accuracy scores of the worst, middle and best image pair of Block 4 of the Structural Learning task for the autistic and non-autistic groups

	Autistic (*n* = 25)		Non-autistic (*n* = 28)	
Continuous variable	*M*	±*SD*	*M*	±*SD*
B4 Worst image pair	0.72	0.25	0.81	0.22
B4 Middle image pair	0.85	0.18	0.90	0.14
B4 Best image pair	0.95	0.10	0.98	0.12

#### Test

**Fig. 4:**
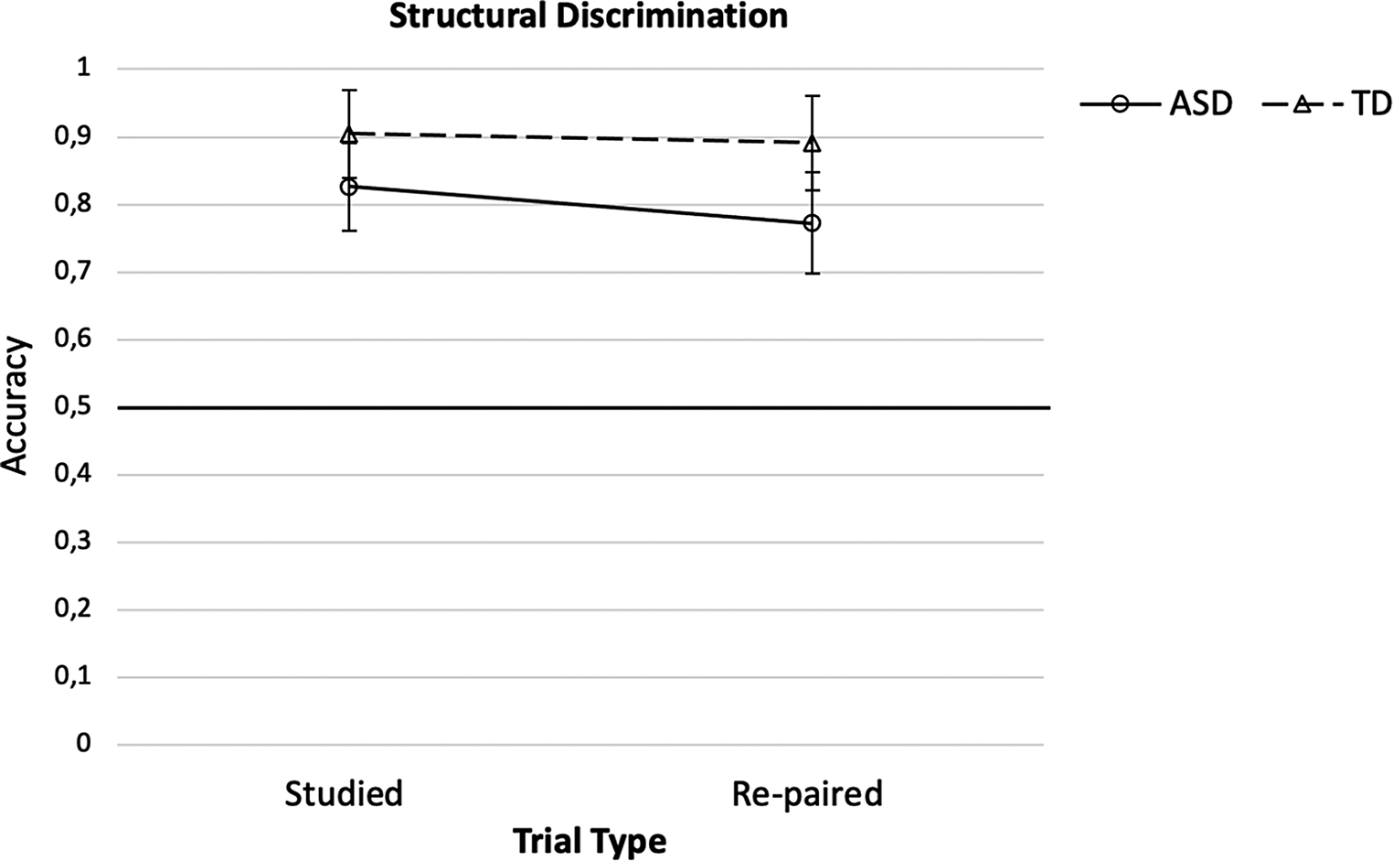
Accuracy scores of the studied and re-paired trials of Block 5 of the Structural Learning task with 95% confidence intervals for the autistic (ASD) and non-autistic (typically developing - TD) groups

To assess the two groups` difference between studied and re-paired trials in Block 5 of the Structural Learning task, ratio scores (re-paired/(re-paired + studied)) were calculated. The data are presented in Fig. 4. Ratio scores did not differ significantly between groups (autistic: *M* = 0.48, *SD* = ± 0.08; non-autistic: *M* = 0.50, *SD* = ± 0.04; *U* = 348.00, *p* = .487, *r* = .004). Comparing participants` (autistic – ASD, non-autistic – typically developing *-* TD) performance on studied and re-paired trials directly using a 2 (Group [ASD, TD]) x 2 (Trial type [studied, re-paired]) repeated measures ANOVA revealed a significant main effect of Group, *F*(1) = 4.70, *p* = .035, η_p_^2^ = 0.08, with higher performance for the TD compared to the ASD group, but no significant main effect of Trial type, *F*(1) = 2.01, *p* = .163, η_p_^2^ = 0.04, GGC, and no significant Group* Trial type interaction, *F*(1) = 0.74, *p* = .395, η_p_^2^ = 0.01, GGC. These results indicate that the TD group showed better performance than the autistic group, independent of the trial type.

#### Relation to Executive Functions

The groups differed significantly in the BRIEF Total Score, *U* = 44.00, *p* < .001, *r* = .73, with better executive functions indicated by lower scores according to parent report for the non-autistic (*M* = 46.72, ±*SD* = 10.83) compared to the autistic group (*M* = 70.88, ±*SD* = 10.99). Due to this difference in the covariate, requirements for analyses with an ANCOVA were not met. Bivariate correlation analysis across the combined sample of autistic and non-autistic participants showed a significant negative correlation between the BRIEF Total Score and performance on re-paired trials in Block 5, *r* = − .32, *p* = .025, indicating that more difficulties in executive functions were associated with lower performance in the re-paired test trials. There was no significant correlation, however, a trend was found between the BRIEF Total Score and the performance on studied trials, *r* = − .25, *p* = .086. Bivariate correlation analysis across the sub-samples of autistic and non-autistic participants showed no significant correlations between the BRIEF Total Score and performance on studied trials (autistic: *r* = .05, *p* = .811; non-autistic: *r* = − .36, *p* = .075), however, there was a trend for the non-autistic group with a larger effect size, and no significant correlation between the BRIEF Total Score and performance on re-paired trials (autistic: *r* = − .07, *p* = .750; non-autistic: *r* = − .32, *p* = .118). Regression analyses revealed no significant difference between the groups’ correlations between the BRIEF Total Score and the performance on studied (*R* = .36, *p*_Interaction_ =.206) or re-paired trials (*R* = .38, *p*_Interaction_ =.463).

## Discussion

The aim of the present study was to examine whether the finding of Structural Learning difficulties in autistic compared to non-autistic adults) can be replicated in a well-matched sample of autistic and non-autistic children. As explained in detail below, this was only partly the case. To fulfil the aim, we analyzed the data of 25 matched autistic and 28 non-autistic children on Simple Discrimination and Structural Learning tasks of Ring et al. (2017).

Hypothesis 1 stating that autistic compared to non-autistic children would show lower learning performance in the Structural Learning task was confirmed only for Block 5 where autistic children showed significantly lower performance compared to the non-autistic group, indicating that autistic children showed difficulties with a Structural Learning task. Hypothesis 2 stating that autistic and non-autistic children would not differ in their learning performance on Simple Discriminations was confirmed for Blocks 2 to 5 on the Simple Discrimination task while autistic compared to non-autistic children showed lower performance in Block 1. The same results for both hypotheses were found for the analysis of the data of all participants tested. The results indicate that both groups were able to learn to discriminate between simple stimuli and they suggest that the between-group difference in performance in Structural Learning cannot be explained by a diminished ability of the autistic group to discriminate between simple stimuli. In this regard the findings are consistent with the findings of Ring et al. (2017) and support the conjecture that atypical hippocampal functioning might play an important role in the etiology of autism (Cooper et al., 2017; Haznedar et al., 2000; Nicolson et al., 2006; Schumann et al., 2004; Solomon et al., 2015). However, differently from Ring et al.’s (2017) finding of a lower performance of autistic compared to non-autistic adults across all five blocks of Structural Learning, we only observed this difference in performance in Block 5. This difference in results could be due to a ceiling effect in the current study, since both groups performed with a high level of accuracy across blocks, making it harder to detect between-group differences. In addition, when directly comparing performance between the studies by inspecting mean values, it can be seen that autistic children in the present study performed better than the autistic adults in Ring et al. (2017) across the blocks, while the non-autistic groups did not differ much in their performance. The difference in performance observed between the autistic children and adults is unlikely to be due to differences in FIQ, since the groups in the two studies were very similar in this respect.

Age could be a relevant factor in explaining the differences in results between studies. It is possible that autistic children and adults may have used different learning strategies for the Structural Learning task which could be due to developmental hippocampal differences. There is considerable evidence indicating structural and functional changes in the hippocampus and fronto-hippocampal circuitry (Lee et al., 2017) especially during the transition from childhood to adolescence (Botdorf et al., 2022) and from adulthood to old age (Langnes et al., 2019). These transitions are accompanied by alterations in functions such as memory (Malykhin et al., 2024), and spatial navigation (Sodoma et al., 2021), suggesting that there might also be similar age-related changes in the functions investigated here. In the present context, transitions in later life may be just as important as those in childhood and adolescence because of the growing body of evidence that younger autistic individuals cognitively resemble older non-autistic persons (‘the ageing analogy’, Bowler, 2007; Ring et al., 2017) and that autism is a ‘different sort of ageing’, (Roestorf, 2018). These considerations point to the need for longitudinal life-span studies.

A surprising result which again could be related to strategy use is the finding that both groups in the current study performed above chance not only in all five blocks of the Structural Learning task, but also contrary to the prediction and Hypothesis 4 in all three discriminations in Block 4, indicating that both groups demonstrated a capacity for Structural Learning. This result was true for the data of all the participants tested here and contrasts with the result of Ring et al. (2017), who found that autistic but not non-autistic adults performed at chance in their worst discrimination in Block 4. The finding also contradicts Derwent’s (2018) findings, where autistic compared to non-autistic children showed performance at chance level in Block 4. The low correlation between executive function scores and task performance predicted by Hypothesis 3 makes an explanation in terms of the compensatory use of executive functions unlikely. However, whereas Ring et al.’s (2017) adults showed undiminished executive functions on the CTT (D’Elia et al., 1996), the autistic children tested here showed lower executive functions in the BRIEF (Gioia et al., 2000) parent report both in the matched and the total dataset. Therefore, it cannot be ruled out that the difference in performance in the Structural Learning task found between the groups in the current study was influenced by this difference in executive functions. More research including functional imaging studies is needed to clarify this. The work by Cooper et al. (2017) on episodic memory in autistic adults using functional imaging gives a hint in this direction showing reduced connectivity of the fronto-hippocampal network compared to non-autistic adults. It would be important to find out whether a similar reduction in fronto-hippocampal connectivity would be the case in autistic children given the behavioral differences between the autistic children tested here and Ring et al.’s (2017) autistic adults.

Hypothesis 5 stating that autistic compared to non-autistic children would show higher performance on re-paired as opposed to studied trials in Block 5 of the Structural Learning task was not confirmed by the present data; the ratio scores of the groups did not differ. Independent of trial type, the non-autistic group showed a better performance compared to the autistic group. The same results were found for the analysis of the data of all participants tested. Therefore, the results contradict the findings of Ring et al. (2017) who found that autistic compared to non-autistic adults showed a better performance on re-paired as opposed to studied trials. We assumed that as in the study from Ring et al. (2017) a reduced learning of the structural arrangement of the mirror images presented in the Structural Learning task in the autistic group would lead to a better performance on re-paired mirror images in this group. We may not have found this better performance on re-paired trials in the autistic group because the autistic children tested here did not show a reduced learning performance in Blocks 1 to 4 of the Structural Learning task unlike the autistic group in the study from Ring et al. (2017). Alternatively, when presented with the re-paired trials, the children in this study may not have used the two-routes strategy to a correct answer described by Ring et al. (2017)^1^, but a different less successful strategy, with the result that the autistic children did not perform better in re-paired as opposed to studied trials.

Executive functions might also have played a role here, as they have been found to influence performance on a hippocampally-dependent task (transverse patterning) in healthy ageing adults (Gracian et al., 2016). Here, executive function difficulties correlated negatively with performance on re-paired trials but not the performance on studied trials for the entire sample. It is conceivable that the characteristics of particular executive functions may determine the extent to which the two-routes strategy to a correct answer referred to above is used and, thus, how successfully the re-paired trials can be solved. In this regard, it is unclear to what extent performance on re-paired trials in the autistic group was affected by difficulties in frontal lobe executive processes and to what extent by hippocampal-related atypicalities associated with difficulties in Structural Learning. Unlike either the present study or that of Ring et al. (2017), Derwent (2018) found no differences between groups in the studied and repaired trials, with their autistic group performing at chance in the re-paired trials. Executive functions were not measured in that study.

The following limitations of the current study need to be considered. The between-group difference in executive functions may have influenced the results. Therefore, future studies should match the groups on executive functions to prevent this. In addition, unlike in Ring et al. (2017) where executive functions were measured directly using the Color Trials Test (D’Elia et al., 1996), the present study used the BRIEF (Gioia et al., 2000), a third-person assessment questionnaire. The use of this instrument might in part explain differences between studies and should, therefore, be considered carefully in future studies (see review by Kenworthy et al., 2008 for a discussion of this topic). Furthermore, groups in the current study differed in the sense that non-autistic participants with co-occurring disorders were excluded from the study whereas many of the autistic participants had additional psychiatric disorders and one took psychotropic medication, which may have influenced the results. Since comorbid psychiatric disorders and the associated use of psychotropic medication are typical for autistic patients, subjects were not excluded from the study in order to ensure a higher generalizability of the results. Another factor that may at least have influenced the correlations might be a power issue due to sample size. Correlations between Structural Learning and BRIEF were significant for the total sample but when splitting the groups there was only a trend for the non-autistic group. Despite our greatest efforts it was not possible to recruit a larger well-matched sample of participants. Finally, it might be argued that we should have corrected the alpha level for multiple comparisons on the same data. However, in line with current recommendations (García-Pérez, 2023), we did not adjust alpha levels, as statistical claims are intended for independent tests where no omnibus tests (such as the repeated-measures ANOVA used here) were available.

The current findings considered in conjunction with those of Ring et al. (2017), by suggesting that autistic children to some extent show difficulties in learning specific structural aspects of configural stimuli, have important implications for future research and interventions with autistic individuals. As already mentioned, life-span longitudinal studies are needed to establish developmental trajectories in the capacity for learning the structural aspects of configural stimuli. Further research is also needed into how Structural Learning ability associates with other hippocampally-mediated functions such as episodic memory and spatial navigation. Studies are also needed to assess the extent and underlying mechanisms of difficulties in Structural Learning capacity associate with aspects of the clinical picture of autism, such as social-communication difficulties and restricted and repetitive behaviors. The discovery of such associations would pave the way for the development of training programs which, because they would be aimed at more abstract, higher-level hippocampal functions, have the potential to have widespread beneficial effects beyond those of current programs that target specific areas of difficulty such as spatial navigation, which requires the ability to differentiate the structural arrangements of components of navigational cues (Diersch & Wolbers, 2019; Lövdén et al., 2012). It is possible to speculate that negotiating social and communicational situations might require a similar ability to respond differently to complex situations that have the same elements but are arranged differently. Envisaging a possible connection between restricted and repetitive behaviors and structural learning is more difficult, but again, we can speculate that if such behaviors are an indirect consequence of other, more directly hippocampally-mediated such as structural learning, then interventions aimed at that might have beneficial knock-on effects. Such speculations would need to be tested empirically.

Taken together, the results suggest that autistic children were able to demonstrate Structural Learning, but showed diminished levels of performance on this measure compared to non-autistic children. Since the processes embodied in Structural Learning tasks are fundamental for spatial navigation (e.g. accurate discrimination between different spatial or temporal configurations of scene components), learning, and episodic memory (flexible re-combination of elements of experience), all of which enable us to orientate and interact successfully in the environment and social world, the present results imply that the ASD clinical picture is associated *inter alia* with difficulties in this domain-general cognitive process of Structural Learning. Its association with atypical hippocampal functioning, demonstrated by non-human animal studies supports, at least in part, a role for hippocampal dysfunction in the clinical picture of ASD. However, since the autistic and non-autistic children differed in their executive functions, it cannot be ruled out that, in addition to atypical hippocampal functioning, atypicalities in the frontal lobe executive system also contribute to the difficulties seen in Structural Learning in autistic children.

## Footnotes

1 On each repaired trial there are two possible routes to a correct answer instead of just one in the studied trials because information from two previously studied trials is crossed. For example, to perform well on a studied trial such as black/white versus white/black (Fig. 1, Column I Row A), the participant needs to know which configuration is correct, that is, that black needs to be on the left side of white. However, to make the correct choice on a repaired trial such as black/white versus striped/white (Fig. 1, Column I Row D), the participant may either remember that black/white rather than white/black (Fig. 1, Column I Row A) is correct, or they may remember that white/striped rather than striped/white is correct (Fig. 1 Column I Row B). Via such a strategy ASD individuals would have been able to increase their performance to above 2/3 of the trials.

## References

[CR49] Aggleton, J. P., Albasser, M. M., Aggleton, D. J., Poirier, G. L., & Pearce, J. M. (2010). Lesions of the rat perirhinal cortex spare the acquisition of a complex configural visual discrimination yet impair object recognition. *Behavioral Neuroscience*, *124*(1), 55–68. 10.1037/a0018320 PMC2834571 20141280

[CR50] Aggleton, J. P., Poirier, G. L., Aggleton, H. S., Vann, S. D., & Pearce, J. M. (2009). Lesions of the fornix and anterior thalamic nuclei dissociate different aspects of hippocampal-dependent spatial learning: Implications for the neural basis of scene learning. *Behavioral Neuroscience*, *123*(3), 504–519 10.1037/a0015404 19485556

[CR51] Aggleton, J. P., Sanderson, D. J., & Pearce, J. M. (2007). Structural learning and the hippocampus. *Hippocampus*, *17*(9), 723–734. 10.1002/hipo.20323 17598160

[CR1] Bachevalier, J. (1994). Medial temporal lobe structures and autism: A review of clinical and experimental findings. *Neuropsychologia*, *32*(6), 627–648. 8084420 10.1016/0028-3932(94)90025-6

[CR2] Bachevalier, J. (1996). Brief report: Medial temporal lobe and autism: A putative animal model in primates. *Journal of Autism and Developmental Disorders*, *26*(2), 217–220. 8744488 10.1007/BF02172015

[CR3] Bölte, S., Rühl, D., Schmötzer, G., & Poustka, F. (2006). ADI-R Diagnostisches Interview für Autismus-Revidiert. Deutsche Fassung des Autism Diagnostic Interview-Revised (ADI-R) von Michael Rutter. *Ann LeCouteur und Catherine Lord. Bern: Huber*.

[CR52] Botdorf, M., Canada, K. L., & Riggins, T. (2022). A meta‐analysis of the relation between hippocampal volume and memory ability in typically developing children and adolescents. *Hippocampus*, *32*(5), 386–400. 10.1002/hipo.23414 PMC9313816 35301771

[CR4] Boucher, J., & Warrington, E. K. (1976). Memory deficits in early infantile autism: Some similarities to the amnesic syndrome. *British Journal of Psychology*, *67*(1), 73–87. 1268453 10.1111/j.2044-8295.1976.tb01499.x

[CR5] Bowler, D., Gaigg, S., & Lind, S. (2011). *Memory in autism: Binding, self and brain. In: Roth, I. & Rezaie, P. (Eds.), Researching the autism spectrum: contemporary perspectives. (pp. 316-346). Cambridge: Cambridge University Press.*

[CR53] Bowler, D. M. (2007). *Autism spectrum disorders: Psychological theory and research*. Wiley.

[CR6] Cooper, R. A., Richter, F. R., Bays, P. M., Plaisted-Grant, K. C., Baron-Cohen, S., & Simons, J. S. (2017). Reduced hippocampal functional connectivity during episodic memory retrieval in autism. *Cerebral Cortex*, *27*(2), 888–902. 28057726 10.1093/cercor/bhw417 PMC5390398

[CR7] DATAtab Team (2023). *DATAtab: Online Statistics Calculator* [Software]. DATAtab e.U. https://datatab.de/

[CR8] D’Elia, L. F., Satz, P., Uchiyana, C. L., & White, T. (1996). *Color trails test. Professional Manual*. Psychological Assessment Resources.

[CR9] DeLong, G. R. (1992). Autism, amnesia, hippocampus, and learning. *Neuroscience & Biobehavioral Reviews*, *16*(1), 63–70. 1553107 10.1016/s0149-7634(05)80052-1

[CR10] Derwent, C. T. (2018). *Relational memory in children with autism spectrum disorder and reduced language* [PhD Thesis]. City, University of London.

[CR54] Desaunay, P., Briant, A. R., Bowler, D. M., Ring, M., Géradin, P., Baleyte, J.-M., Guénolé, F., Eustache, F., Parienti, J.-J., & Guillery-Girard, B. (2020). Memory in autism spectrum disorder: A meta-analysis of experimental studies. *Psychological Bulletin*, *146*(5), 377–410. 10.1037/bul0000225 32191044

[CR55] Diersch, N., & Wolbers, T. (2019). The potential of virtual reality for spatial navigation research across the adult lifespan. *Journal of Experimental Biology*, *222*(Suppl_1). 10.1242/jeb.187252 30728232

[CR11] Ellis Weismer, S., Kaushanskaya, M., Larson, C., Mathée, J., & Bolt, D. (2018). Executive function skills in school-age children with autism spectrum disorder: Association with language abilities. *Journal of Speech Language and Hearing Research*, *61*(11), 2641–2658. 10.1044/2018_JSLHR-L-RSAUT-18-0026 PMC6693571 30418493

[CR12] Friedman, L., & Sterling, A. (2019). A review of language, executive function, and intervention in autism spectrum disorder. *Seminars in Speech and Language*, *40*(04), 291–304. 31311054 10.1055/s-0039-1692964 PMC7012379

[CR13] García-Pérez, M. A. (2023). Use and misuse of corrections for multiple testing. *Methods in Psychology*, *8*, 100120. 10.1016/j.metip.2023.100120

[CR14] Gioia, G. A., Isquith, P. K., Guy, S. C., & Kenworthy, L. (2000). *Behavior rating inventory of executive function: BRIEF*. Psychological Assessment Resources Odessa, FL.

[CR56] Gracian, E. I., Osman, D. C., & Mosack, K. E. (2016). Transverse patterning, aging, and neuropsychological correlates in humans. Hippocampus, *26*(12), 1633–1640. 10.1002/hipo.22662 27658032

[CR15] Haznedar, M. M., Buchsbaum, M. S., Wei, T. C., Hof, P. R., Cartwright, C., Bienstock, C. A., & Hollander, E. (2000). Limbic circuitry in patients with autism spectrum disorders studied with positron emission tomography and magnetic resonance imaging. *American Journal of Psychiatry*, *157*(12), 1994–2001. 11097966 10.1176/appi.ajp.157.12.1994

[CR16] Hill, E. L. (2004). Executive dysfunction in autism. *Trends in Cognitive Sciences*, *8*(1), 26–32. 14697400 10.1016/j.tics.2003.11.003

[CR17] Hosenbocus, S., & Chahal, R. (2012). A review of executive function deficits and pharmacological management in children and adolescents. *Journal of the Canadian Academy of Child and Adolescent Psychiatry*, *21*(3), 223. 22876270 PMC3413474

[CR57] Langnes, E., Vidal-Piñiero, D., Sneve, M. H., Amlien, I. K., Walhovd, K. B., & Fjell, A. M. (2019). Development and decline of the hippocampal long-axis specialization and differentiation during encoding and retrieval of episodic memories. *Cerebral Cortex*, *29*(8), 3398–3414. 10.1093/cercor/bhy209 30272128

[CR58] Lee, J.K., Johnson, E.G., Ghetti, S. (2017). Hippocampal Development: Structure, Function and Implications. In: D. Hannula, & M. Duff (Eds.), *The Hippocampus from Cells to Systems*. Springer, Cham.

[CR20] Lind, S. E., Bowler, D. M., & Raber, J. (2014). Spatial navigation, episodic memory, episodic future thinking, and theory of mind in children with autism spectrum disorder: Evidence for impairments in mental simulation? *Frontiers in Psychology*, *5*, 1411. 25538661 10.3389/fpsyg.2014.01411 PMC4256988

[CR21] Lind, S. E., Williams, D. M., Raber, J., Peel, A., & Bowler, D. M. (2013). Spatial navigation impairments among intellectually high-functioning adults with autism spectrum disorder: Exploring relations with theory of mind, episodic memory, and episodic future thinking. *Journal of Abnormal Psychology*, *122*(4), 1189. 24364620 10.1037/a0034819 PMC3906800

[CR19] Li, S., Hu, J., Chang, R., Li, Q., Wan, P., & Liu, S. (2021). Eye movements of spatial Working Memory Encoding in Children with and without autism: Chunking Processing and Reference Preference. *Autism Research: Official Journal of the International Society for Autism Research*, *14*(5), 897–910. 10.1002/aur.2398 32959979 10.1002/aur.2398

[CR59] Livingston, L. A., & Happé, F. (2017). Conceptualising compensation in neurodevelopmental disorders: Reflections from autism spectrum disorder. *Neuroscience & Biobehavioural Reviews*, *80*, 729–742. 10.1016/j.neubiorev.2017.06.005 PMC7374933 28642070

[CR60] Lövdén, M., Schaefer, S., Noack, H., Bodammer, N. C., Kühn, S., Heinze, H.-J., Düzel, E., Bäckman, L., & Lindenberger, U. (2012). Spatial navigation training protects the hippocampus against age-related changes during early and late adulthood. *Neurobiology of Aging*, *33*(3), 620.e9–620.e22. 10.1016/j.neurobiolaging.2011.02.013 21497950

[CR61] Malykhin, N., Pietrasik, W., Hoang, K. N., & Huang, Y. (2024). Contributions of hippocampal subfields and subregions to episodic memory performance in healthy cognitive aging. *Neurobiology of Aging*, *133*, 51–66. 10.1016/j.neurobiolaging.2023.10.006 37913626

[CR22] Marini, A., Ferretti, F., Chiera, A., Magni, R., Adornetti, I., Nicchiarelli, S., Vicari, S., & Valeri, G. (2019). Episodic future thinking and narrative discourse generation in children with Autism Spectrum disorders. *Journal of Neurolinguistics*, *49*, 178–188.

[CR23] Marsh, L., Pearson, A., Ropar, D., & Hamilton, A. (2013). Children with autism do not overimitate. *Current Biology*, *23*(7), R266–R268. 23578869 10.1016/j.cub.2013.02.036

[CR24] Mayes, S. D., & Calhoun, S. L. (2007). Learning, attention, writing, and processing speed in typical children and children with ADHD, autism, anxiety, depression, and oppositional-defiant disorder. *Child Neuropsychology*, *13*(6), 469–493. 17852125 10.1080/09297040601112773

[CR25] Naito, M., Hotta, C., & Toichi, M. (2020). Development of episodic memory and foresight in high-functioning preschoolers with ASD. *Journal of Autism and Developmental Disorders*, *50*(2), 529–539. 10.1007/s10803-019-04274-9 31745700 10.1007/s10803-019-04274-9

[CR26] Nicolson, R., DeVito, T. J., Vidal, C. N., Sui, Y., Hayashi, K. M., Drost, D. J., Williamson, P. C., Rajakumar, N., Toga, A. W., & Thompson, P. M. (2006). Detection and mapping of hippocampal abnormalities in autism. *Psychiatry Research: Neuroimaging*, *148*(1), 11–21. 10.1016/j.pscychresns.2006.02.005 17056234

[CR27] O’Brien, G., & Pearson, J. (2004). Autism and learning disability. *Autism*, *8*(2), 125–140. 15165430 10.1177/1362361304042718

[CR28] O’Keefe, J., & Nadel, L. (1978). *The hippocampus as a cognitive map*. Clarendon Press; Oxford University.

[CR29] Petermann, F. (2017). *WISC-V: Wechsler Intelligence Scale for Children—Fifth Edition Von David Wechsler*. Pearson.

[CR30] Poustka, L., Rühl, D., Feineis-Matthews, S., Bölte, S., Poustka, F., & Hartung, M. (2015). ADOS-2: Diagnostische Beobachtungsskala für Autistische Störungen–2. *Bern: Huber*.

[CR31] Ring, M., Derwent, C. L., Gaigg, S. B., & Bowler, D. M. (2017). Structural learning difficulties implicate altered hippocampal functioning in adults with Autism Spectrum Disorder. *Journal of Abnormal Psychology, 126*(6), 793-804. 10.1037/abn0000277 28557507

[CR62] Roestorf, A. (2018). *Ageing, cognition and quality of life in autism spectrum disorder: cross-sectional and longitudinal studies* [Unpublished Doctoral thesis, City, University of London].

[CR35] Rubin, R. D., Watson, P. D., Duff, M. C., & Cohen, N. J. (2014). The role of the hippocampus in flexible cognition and social behavior. *Frontiers in Human Neuroscience*, *8*, 742. 25324753 10.3389/fnhum.2014.00742 PMC4179699

[CR36] Rudy, J. W., & Sutherland, R. J. (1995). Configural association theory and the hippocampal formation: An appraisal and reconfiguration. *Hippocampus*, *5*(5), 375–389. 8773252 10.1002/hipo.450050502

[CR37] Sanderson, D. J., Pearce, J. M., Kyd, R. J., & Aggleton, J. P. (2006). The importance of the rat hippocampus for learning the structure of visual arrays. *European Journal of Neuroscience*, *24*(6), 1781–1788. 17004941 10.1111/j.1460-9568.2006.05035.x

[CR38] Schiller, D., Eichenbaum, H., Buffalo, E. A., Davachi, L., Foster, D. J., Leutgeb, S., & Ranganath, C. (2015). Memory and space: Towards an understanding of the cognitive map. *Journal of Neuroscience*, *35*(41), 13904–13911. 26468191 10.1523/JNEUROSCI.2618-15.2015 PMC6608181

[CR39] Schumann, C. M., Hamstra, J., Goodlin-Jones, B. L., Lotspeich, L. J., Kwon, H., Buonocore, M. H., Lammers, C. R., Reiss, A. L., & Amaral, D. G. (2004). The amygdala is enlarged in children but not adolescents with autism; the hippocampus is enlarged at all ages. *Journal of Neuroscience*, *24*(28), 6392–6401. 15254095 10.1523/JNEUROSCI.1297-04.2004 PMC6729537

[CR63] Sodoma, M. J., Cole, R. C., Sloan, T. J., Hamilton, C. M., Kent, J. D., Magnotta, V. A., & Voss, M. W. (2021). Hippocampal acidity and volume are differentially associated with spatial navigation in older adults. *Neuroimage*, *245,* 118682. 10.1016/j.neuroimage.2021.118682 PMC8867536 34728245

[CR40] Solomon, M., Ragland, J. D., Niendam, T. A., Lesh, T. A., Beck, J. S., Matter, J. C., Frank, M. J., & Carter, C. S. (2015). Atypical learning in autism spectrum disorders: A functional magnetic resonance imaging study of transitive inference. *Journal of the American Academy of Child & Adolescent Psychiatry*, *54*(11), 947–955. 26506585 10.1016/j.jaac.2015.08.010 PMC4624100

[CR41] Sutherland, R. J., & Rudy, J. W. (1989). Configural association theory: The role of the hippocampal formation in learning, memory, and amnesia. *Psychobiology*, *17*(2), 129–144.

[CR64] Vargha-Khadem, F., Gadian, D. G., Watkins, K. E., Conelly, A., Van Paesschen, W., & Mishkin, M. (1997). Differential effects of early hippocampal pathology on episodic and semantic memory. *Science*, *277*(5324), 376–380. 10.1126/science.277.5324.376 9219696

[CR43] Vogan, V. M., Morgan, B. R., Smith, M. L., & Taylor, M. J. (2019). Functional changes during visuo-spatial working memory in autism spectrum disorder: 2-year longitudinal functional magnetic resonance imaging study. *Autism*, *23*(3), 639–652. 29595335 10.1177/1362361318766572

[CR44] Waterhouse, L., Fein, D., & Modahl, C. (1996). Neurofunctional mechanisms in autism. *Psychological Review*, *103*(3), 457. 8759044 10.1037/0033-295x.103.3.457

[CR45] World Health Organization. (2004). *ICD-10: International Statistical classification of diseases and related health problems: Tenth revision* (2nd ed.).). World Health Organization. 3376487

[CR46] Zeidan, J., Fombonne, E., Scorah, J., Ibrahim, A., Durkin, M. S., Saxena, S., Yusuf, A., Shih, A., & Elsabbagh, M. (2022). Global prevalence of autism: A systematic review update. *Autism Research*, *15*(5), 778–790. 10.1002/aur.2696 35238171 10.1002/aur.2696 PMC9310578

[CR47] Zhang, M., Jiao, J., Hu, X., Yang, P., Huang, Y., Situ, M., Guo, K., Cai, J., & Huang, Y. (2020). Exploring the spatial working memory and visual perception in children with autism spectrum disorder and general population with high autism-like traits. *PloS One*, *15*(7), e0235552. 10.1371/journal.pone.0235552 32645114 10.1371/journal.pone.0235552 PMC7347168

[CR48] Zhang, X., Qiu, Y., Li, J., Jia, C., Liao, J., Chen, K., Qiu, L., Yuan, Z., & Huang, R. (2022). Neural correlates of transitive inference: An SDM meta-analysis on 32 fMRI studies. *NeuroImage*, 119354. 10.1016/j.neuroimage.2022.119354 35659997

